# Allelopathic Activity of a Novel Compound, Two Known Sesquiterpenes, and a C_13_ *Nor*-Isopenoid from the Leave of *Croton oblongifolius* Roxb. for Weed Control

**DOI:** 10.3390/plants12193384

**Published:** 2023-09-25

**Authors:** Seinn Moh Moh, Shunya Tojo, Toshiaki Teruya, Hisashi Kato-Noguchi

**Affiliations:** 1 Department of Applied Biological Science, Faculty of Agriculture, Kagawa University, Miki 761-0795, Kagawa, Japan; hanmohmohyau@gmail.com; 2 The United Graduate School of Agricultural Sciences, Ehime University, Matsuyama 790-8566, Ehime, Japan; 3 Graduate School of Engineering and Science, University of the Ryukyus, 1 Senbaru, Nishihara 903-0213, Okinawa, Japan; k218370@eve.u-ryukyu.ac.jp; 4 Faculty of Education, University of the Ryukyus, 1 Senbaru, Nishihara 903-0213, Okinawa, Japan; t-teruya@edu.u-ryukyu.ac.jp

**Keywords:** *Croton oblongifolius*, allelopathic potential, allelopathic substances, (3*R*,6*R*,7*E*)-3-hydroxy-4-7-megastigmadien-9-one, 2-hydroxy alpinolide, alpinolide, epialpinolide

## Abstract

Investigation of allelopathic substances from herbal plants may lead to the development of allelochemical-based natural herbicides. *Croton oblongifolius* (Roxb.) is a well-known herbal plant with a long history of being used for traditional medicines and for being the source of a diverse range of bioactive compounds. This plant has been reported to have allelopathic potential; however, its allelopathic-related substances have not yet been described. Therefore, we conducted this investigation to explore the allelopathic substances from the leaves of *C. oblongifolius*. Aqueous methanol extracts of *C. oblongifolius* leaves exhibited significant growth inhibitory potential against four test plants (monocot barnyard grass and timothy, and dicot cress and lettuce). The leaf extracts were purified in various chromatographic steps and yielded four active compounds identified as (3*R*,6*R*,7*E*)-3-hydroxy-4-7-megastigmadien-9-one (I), 2-hydroxy alpinolide (a novel compound) (II), alpinolide (III), and epialpinolide (IV) via an analysis of the spectral data. These identified compounds significantly restricted the seedling growth of cress. The concentration necessary for 50% growth reduction of the cress seedlings varied from 0.15 to 0.24 mM for (3*R*,6*R*,7*E*)-3-hydroxy-4-7-megastigmadien-9-one, 0.04 to 0.11 mM for 2-hydroxy alpinolide, 0.07 to 0.12 mM for alpinolide, and 0.09 to 0.16 mM for epialpinolide. Therefore, the leaf extracts of *C. oblongifolius* and the characterized compounds have the potential to be used as weed-suppressive resources for natural weed control.

## 1. Introduction

Plant allelopathy has been extensively explored for a long time as an alternative weed management strategy to reduce the reliance on synthetic herbicides and to reduce the negative effects of herbicides for sustainable weed management [1,2,3]. For effective weed management, most farmers frequently depend on synthetic herbicides [4]. However, the improper use of artificial chemicals for weed and pest management in agro-ecosystems has resulted in increases in environmental pollution and health hazards, herbicide tolerance in weeds, and contamination of agricultural commodities by herbicide residues [5]. Therefore, it has become essential to develop alternative and eco-friendly weed management strategies.

Allelopathy is becoming a more popular approach as a sustainable weed management method for overcoming the problems of environmental contamination and herbicide resistance [6]. Such a method can combat harmful weeds and improve crop productivity and soil quality when allelopathic plants are used for mulching, cover crops, residue incorporation, crop rotation, and plant extracts with phytochemical potential [5,6]. A large number of plant species release secondary metabolites (known as allelochemicals) during the process of allelopathy [7]. These secondary metabolites are biologically potent compounds that can be used as allelochemicals against weeds, herbs, microorganisms, and nematodes. In addition, phenolic compounds that include terpenoids and isothiocynates obtained from various crop species can be used as weedicides, herbicides, and fungicides, and polysulfides obtained from garlic can be used as a nematocide [8]. Therefore, natural compounds produced by plants have received much attention during the past two decades [9,10]. For instance, Kato-Noguchi and Ota (2013) [11] documented that the allelochemicals momilactones A and B from rice secretory fluid and root exudates showed biological activity against monocot weed species (barnyard grass, crabgrass, jungle rice, timothy, and Italian ryegrass) and dicot plant species (arabidopsis, lettuce, cress, and alfalfa), and Kyaw et al. (2022) [12] observed that two bioactive substances (3-hydroxy-*α*-ionone and 5-hydroxy3,4-dimethyl-5-pentylfuran-2(5*H*)-one) from *Dregea volubilis* had phytotoxic effects against cress and barnyard grass. Moh et al. (2023) [13] reported the phytotoxic activity of a novel compound, 8-dehydroxy-11*β*-*O*-acetyl-12*β*-*O*-tigloyl-17*β*-marsdenin, identified from *Marsdenia tenacissima*, against the seedling growth of cress. These findings indicate that a variety of plant species, including herbal plants, possess bioactive compounds that can be developed as bioherbicides for natural weed control.

**Figure 1:**
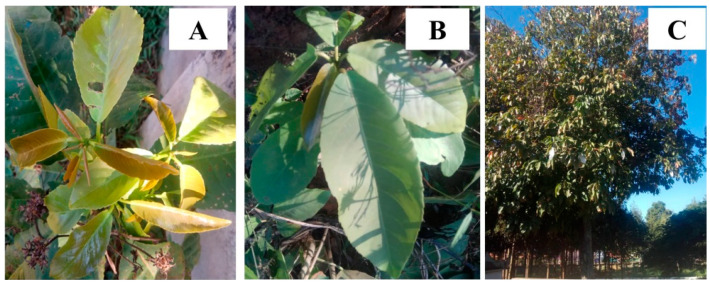
(**A**) Shoot, (**B**) leaf, and (**C**) whole plant of *Croton oblongifolius* Roxb.

The Euphorbiaceae family comprises nearly 1300 species of trees, herbs, and shrubs, commonly found in subtropical and tropical areas around the globe [14]. Several species from the *Croton* genus under this family have been used as folk medicines for treating many diseases in Africa, East and South Asia, and South America [14,15,16]. *Croton oblongifolius* Roxb. (Euphorbiaceae) is a middle-sized tree, commonly distributed in Myanmar, India, Sri Lanka, Nepal, Bhutan, Bangladesh, China, Thailand, Cambodia, and Vietnam [17] (Figure 1). The leaves of this plant are used as a tonic, and the fruits and seeds are used to treat dysmenorrhea and purgatives [18]. In addition, the bark and roots have been used to treat dyspepsia and dysentery [19]. Other studies have reported that the leaves, stem bark, and roots of this plant contain phytochemicals such as monoterpenes, sesquiterpenes, megastigmane glycosides, phenylpropanoids, and diterpenoids (including cembranes, labdanes, cleistanthanes, halimanes, isopimaranes and clerodanes, for instance, nasimalun A) [18,20,21,22,23,24,25]. The biological activities of this plant include anticancer, anti-inflammatory [26,27,28], antibacterial [20], and hepatoprotective [28]. Although the potential allelopathic activity of *C. oblongifolius* has been mentioned by Sothearith et al. (2021) [29], there is no information about its allelopathic compounds. Therefore, the aim of this research was to investigate the allelopathic potential of and related compounds from *C. oblongifolius* leaves. 

## 2. Results

### 2.1. Inhibitory Activity of Croton oblongifolius

**Figure 2:**
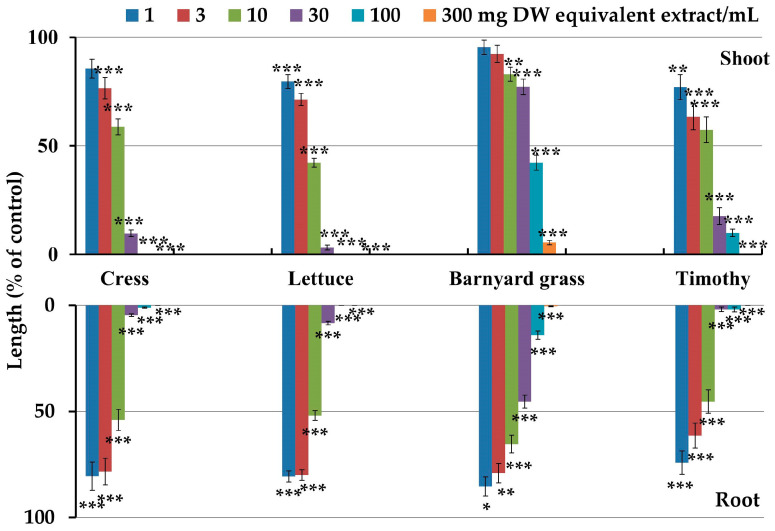
Effects of the *Croton oblongifolius* extracts at the concentrations of 1, 3, 10, 30, 100, and 300 mg DW equivalent extract/mL on the shoot and root growth of the dicot (cress and lettuce) and monocot plants (barnyard grass and timothy). Significant differences among the control and treatments are shown by asterisks: * *p* < 0.05, ** p < 0.01, *** *p* < 0.001.

The leaf extracts of *C. oblongifolius* inhibited the seedling growth of both the dicot (cress and lettuce) and monocot plants (barnyard grass and timothy) at concentrations greater than 10 mg DW equivalent extract/mL except the barnyard grass (*p* < 0.05) (Figure 2). At the concentration of 30 mg DW equivalent extract/mL, the shoot development of cress, lettuce, barnyard grass, and timothy was inhibited to 9.8, 3.2, 77.2, and 17.6%, respectively, of the control shoots, whereas the root growth of these plants was inhibited to 4.6, 8.4, 45.3, and 1.9%, respectively, of the control roots. Additionally, in contrast with the control, 300 mg DW equivalent extract/mL concentration fully suppressed the shoot and root growth of cress, lettuce, and timothy, whereas the shoot and root development of barnyard grass were inhibited to 5.5 and 0.5%, respectively.

The *I*_50_ values of the *C. oblongifolius* extracts for the shoot and root growth of the dicot (cress and lettuce) and monocot plants (barnyard grass and timothy) varied from 4.8 to 65.7 mg DW equivalent extract/mL (Table 1). The *I*_50_ value for the shoot growth of cress (*I*_50_ = 9.4) did not differ significantly from that of its root growth (*I*_50_ = 8.3), and the value for the root growth of lettuce (*I*_50_ = 8.2) was significantly greater than for its shoot growth (*I*_50_ = 5.8), whereas the value for the root growth of barnyard grass (*I*_50_ = 18.2) and timothy (*I*_50_ = 4.8) was significantly less than for their shoot growth, with (*I*_50_ = 65.7) and (*I*_50_ = 7.6), respectively. The values of the correlation coefficient (r) varied from −0.71 to −0.91 among the seedling growth of the representative test plants and *C. oblongifolius* extract concentrations, exhibiting a negative correlation (Table 1).

### 2.2. Identification of the Allelopathic Substances

**Figure 3:**
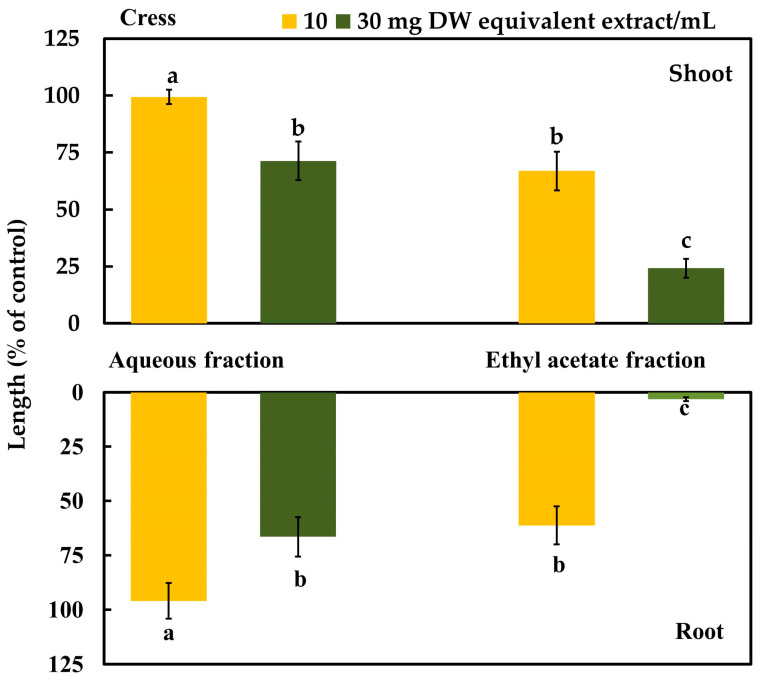
Effects of the aqueous and EtOAc fractions partitioned from the *Croton oblongifolius* extracts on the shoot and root growth of cress. The letters indicate significant variations at less than the 0.05 probability level (Tukey’s test).

The aqueous and ethyl acetate (EtOAc) fractions (partitioned from the aqueous residue of *C. oblongifolius*) had significant dose-dependent, limiting effects on the growth of the cress seedlings (*p* < 0.05) (Figure 3). However, the inhibitory effects of the EtOAc fraction were higher than those of the aqueous fraction at all dosages. From these results, the EtOAc fraction was purified in various chromatographic steps using silica gel, Sephadex LH-20, reverse-phase ODS cartridge, and reverse-phase HPLC. Four active substances were isolated using reverse-phase HPLC and characterized using spectral data.

**Figure 4:**
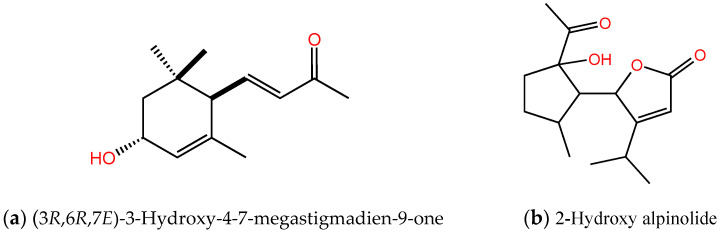

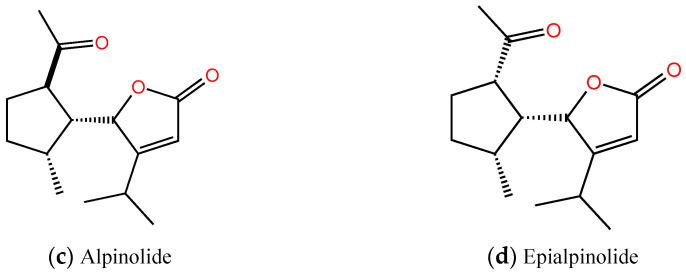
Molecular structures of (**a**) compound I, (**b**) compound II, (**c**) compound III, and (**d**) compound IV from *Croton oblongifolius*.

Compound I was observed as a colorless oil, and its molecular formula was identified as C_13_H_20_O_2_Na through HR-ESIMS *m*/*z* 231.1356 [M+Na]^+^ (calcd for C_15_H_20_O_2_Na, 231.1356). The ^1^H NMR (500 MHz, CDCl_3_) spectrum showed δ_H_ 6.54 (1H, dd, *J* = 15.8, 10.2 Hz), 6.10 (1H, d, *J* = 15.8 Hz), 5.63 (1H, m), 4.27 (1H, m), 2.50 (1H, d, *J* = 10.2 Hz), 2.26 (3H, s), 1.84 (1H, dd, *J* = 13.5, 6.0 Hz), 1.62 (3H, s), 1.40 (1H, dd, *J* = 13.5, 6.3 Hz), 1.03 (3H, s), 0.89 (3H, s). Compound I was characterized as (3*R*,6*R*,7*E*)-3-hydroxy-4-7-megastigmadien-9-one by comparing these spectroscopic data with the data described in a published article [30] (Figure 4a).

Compound II was observed as a colorless oil; [*α*]_D_^23^ = −40.5 (c 0.04, CHCl_3_). The molecular formula was identified as C_15_H_22_O_4_Na through HR-ESIMS *m*/*z* 289.1410 [M+Na]^+^ (calcd for C_15_H_22_O_4_Na, 289.1410) (Figure 4b and Figure S1). The NMR spectra data are described in Table 2 and Figure S2.

Compound III was observed as a colorless oil, and its chemical formula was identified as C_15_H_22_O_3_Na through HR-ESIMS *m*/*z* 273.1461 [M+Na]^+^ (calcd for C_15_H_22_O_3_Na, 273.1461). The ^1^H NMR (500 MHz, CDCl_3_) spectrum showed δ_H_ 5.80 (1H, t, *J* = 1.7 Hz), 5.07 (1H, dd, *J* = 3.4, 1.9 Hz), 3.38 (1H, m), 2.68 (1H, m), 2.67 (1H, m), 2.24 (1H, m), 2.11 (1H, dq, *J* = 2.6, 7.1 Hz), 1.77 (1H, m), 1.58 (1H, m), 1.41 (1H, m), 1.28 (3H, d, *J* = 6.7 Hz), 1.22 (3H, d, *J* = 7.1 Hz), 0.78 (3H, d, *J* = 7.1 Hz). This compound was identified as alpinolide (Figure 4c) after comparing these data with those published by Itokawa et al. (1984) [31].

Compound IV was observed as a colorless oil, and the molecular formula was identified as C_15_H_23_O_3_ by using HR-ESIMS *m*/*z* 251.1642 [M+H]^+^ (calcd for C_15_H**_23_**O_3_, 251.1642). The ^1^H NMR (500 MHz, CDCl_3_) spectrum showed δ_H_ 5.75 (1H, t, *J* = 1.6 Hz), 5.59 (1H, dd, *J* = 4.7, 1.8 Hz), 3.05 (1H, m), 2.54 (1H, m), 2.39 (1H, m), 2.27 (1H, m), 2.23 (3H, s), 2.20 (1H, m), 2.07 (1H, m), 1.84 (1H, m), 1.57 (1H, m), 1.26 (3H, d, *J* = 6.7 Hz), 1.16 (3H, d, *J* = 7.0 Hz), 0.94 (3H, d, *J* = 7.2 Hz). This compound was identified as epialpinolide (Figure 4d) after comparing these data with those in the published literature [32].

### 2.3. Allelopathic Activity of Compounds I, II, III, and IV

**Figure 5:**
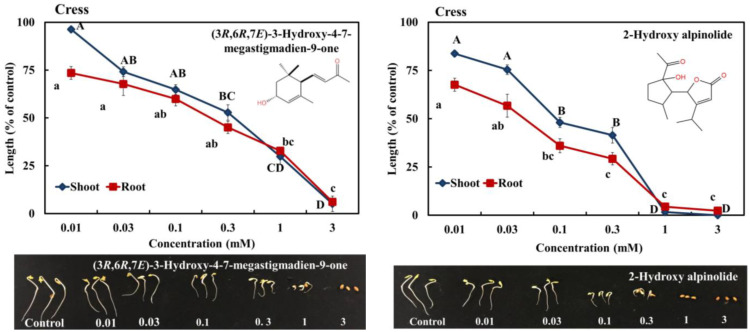

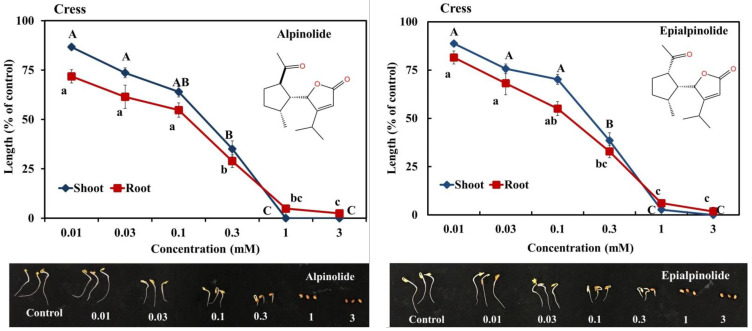
Effects of compounds I, II, III, and IV on the shoot and root growth of cress. The letters show significant differences between the treatments at less than the 0.05 probability level (Tukey’s test).

The four characterized compounds significantly retarded the seedling growth of cress, and the degree of growth suppression activity depended on the compound concentration. Compound I significantly restricted the cress shoots and roots at concentrations greater than 0.3 and 0.1 mM, respectively (*p* < 0.05) (Figure 5). Compound II significantly inhibited the cress shoots and roots at a concentration greater than 0.03 mM (Figure 5). Compounds III and IV significantly suppressed the cress shoots and roots at a concentration greater than 0.1 mM (Figure 5).

The *I*_50_ values for the shoot and root growth of cress ranged from 0.15 to 0.24 mM for compound I, 0.04 to 0.11 mM for compound II, 0.07 to 0.12 mM for compound III, and 0.09 to 0.16 mM for compound IV (Table 3). For compounds I, II, III, and IV, the *I*_50_ values for the cress shoots were 1.6, 2.75, 1.71, and 1.77 times greater than for the roots, respectively. Compound II showed stronger growth inhibitory potential than compounds I, III, and IV.

## 3. Discussion

The leaf extracts of *C. oblongifolius* significantly restricted the seedling growth of the dicot (cress and lettuce) and monocot plants (barnyard grass and timothy) (Figure 2). The correlation coefficient (r) revealed a significant negative relationship between the shoot and root length of the test plants and the concentration of the *C. oblongifolius* extracts (Table 1), showing that the extract concentration affected the growth inhibitory activity. Additionally, the *I*_50_ values for the cress, lettuce, barnyard grass, and timothy shoot and root growth differed, indicating that inhibition via the *C. oblongifolius* extracts is species-dependent (Table 1). Similar outcomes of dose- and species-dependent inhibitory effects have been shown for extracts of *Marsdenia tenacissima*, *Garcinia Xanthochymus*, *Polygonum chinense*, and *Anredera cordifolia* [33,34,35,36]. Thus, the inhibitory activity of the *C. oblongifolius* extracts on the growth of the four examined plant species suggests that its extracts may possess phytotoxic substances.

The EtOAc fraction was separated and loaded on a silica gel column. In each chromatographic step, the biological activities of the active fraction were evaluated in a cress bioassay. The most active fraction was purified by chromatography using Sephadex LH-20, reverse-phase ODS or C18 cartridges, and HPLC, resulting in the isolation of four active compounds (I, II, III, and IV).

Compound I, (3*R*,6*R*,7*E*)-3-hydroxy-4,7-megastigmadien-9-one, is a C_13_ *nor*-isopenoid derivative from carotenoids [30,37]. Compounds under C_13_ *nor*-isopenoid have many pharmacological [38] and allelopathic properties [12,30,39,40]. This compound has also been identified from *Viburnum dilatatum* [41] and *Camellia nitidissima* [38]. However, the present study is the first to document the plant growth restriction activity of (3*R*,6*R*,7*E*)-3-hydroxy-4,7-megastigmadien-9-one from *C. oblongifolius*.

**Figure 6:**
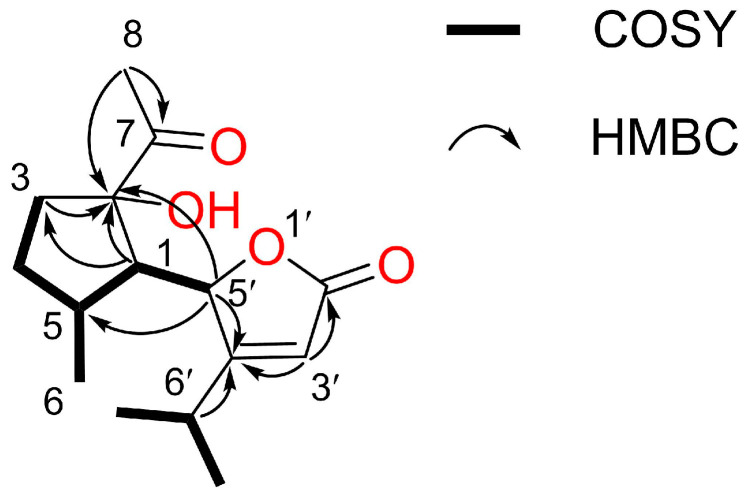
Key 2D NMR (COSY and HMBC) correlation of compound II.

The ^1^H and ^13^C NMR of compound II, as measured in CD_3_OD (500 MHz), showed the presence of four methyl groups at δ_H_ 2.35 (3H, s), 1.28 (3H, d, *J* = 6.6 Hz), 1.16 (3H, d, *J* = 7.0 Hz), and 0.85 (3H, d, *J* = 7.3 Hz); one olefic proton at δ_H_ 5.87 (1H, t, *J* = 1.6 Hz); four methine protons at δ_H_ 5.41 (1H, dd, *J* = 3.8, 1.8 Hz), 2.71 (1H, m), 2.48 (1H, m), and 2.36 (1H, dd, *J* = 7.5, 3.8 Hz); and four methylene protons at δ_H_ 2.47 (1H, m), 2.03 (1H, m), 1.66 (1H, ddd, *J* = 13.7, 9.0, 4.9 Hz), and 1.51 (1H, m) (Table 2) (Figure S2). In the ^13^C NMR spectrum, fifteen carbon signals were observed, including two carbonyl carbons (δ_C_ 214.1 and 175.7), four methyl carbons (δ_C_ 26.9, 22.2, 20.3, and 16.0), one quaternary carbon (δ_c_ 88.7), two olefinic carbons (δ_C_ 182.4 and 115.1), four methine carbons (δ_C_ 83.1, 56.5, 36.1, and 28.6), and two methylene carbons (δ_C_ 36.9 and 32.6). The COSY spectrum of compound II shows two incomplete structures C3-C4-C5(-C6)-C1-C5′ and C7′-C6′-C8′ (Figure 6 and Figure S3). The connectivities of these two incomplete structures were found in the HMBC spectrum to be H1 (δ_H_ 2.36) to C2 (δ_C_ 88.7) and C3 (δ_C_ 36.9); H3 (δ_H_ 2.47) to C2 (δ_C_ 88.7); H8 (δ_H_ 2.35) to C2 (δ_C_ 88.7) and C7 (δ_C_ 214.1); H3′ (δ_H_ 5.87) to C2′ (δ_C_ 175.7) and C4′ (δ_C_ 182.4); H5′ (δ_H_ 5.41) to C2 (δ_C_ 88.7), C5 (δ_C_ 36.1), and C4′ (δ_C_ 182.4); and H6′ (δ_H_ 2.71) to C4′ (δ_C_ 182.4), suggesting one hydroxy group was located at the C2 position (Figure 6 and Figure S4). As a result of its structure, compound II was identified as a novel compound (2-hydroxy alpinolide) and defined as 5-(2-acetyl-2-hydroxy-5-methylcyclopentyl)-4-isopropylfuran-2 (5*H*)-one.

Compounds III (alpinolide) and IV (epialpinolide) are secoguaiane-type sesquiterpenes with an *α* and *β*-unsaturated butenolide. These compounds were isolated for the first time by Itokawa et al. (1984) [31] and Itokawa et al. (1987) [32] from *Alpinia japonica* and *Alpinia intermedia*, respectively. Alpinolide has a similar molecular structure to epialpinolide (Figure 4c,d), except for the presence of *α* and *β* orientation at C-1. Moreover, epialpinolide is an epimer of alpinolide, and the stereochemistry of the methyl ketone group of this compound should be *α* [32]. Thus, the chemical name of alpinolide and epialpinolide is defined as 5-(2-acetyl-5-methylcyclopentyl)-4-isopropylfuran-2(5*H*)-one.

The isolated compounds 2-hydroxyalpinolide, alpinolide, and epialpinolide are a group of secoguaiane-type sesquiterpenes that are the derivatives of the terpenoid. Many researchers have documented the pharmacological and insect-resistant ability of terpenoids from herbal plants [42]. In addition, several compounds under the group of sesquiterpenes possess anticancer [43,44,45] and antibacterial properties [46]. Moreover, sesquiterpene lactone, dehydrozaluzanin C isolated from weeds of the Compositae family, have shown bioherbicidal potential with high inhibitory activity against dicotyledonous plants compared with the commercial herbicide, Logran^®^ [47]. Additionally, the series of sesquiterpenes such as alpinolide, epialpinolide, and 6-hydroxy alpinolide can be found in many plant species of the Zingiberaceae family, and these compounds exhibit many biological activities [31,48,49]. Nonetheless, this study is the first to isolate the phytotoxic compounds with strong inhibitory effects of 2-hydroxy alpinolide, alpinolide, and epialpinolide from *C. oblongifolius*.

The results of the current research show that (3*R*,6*R*,7*E*)-3-hydroxy-4,7-megastigmadien-9-one, 2-hydroxy alpinolide, alpinolide, and epialpinolide significantly restrict the seedling growth of cress (Figure 5). Based on the *I*_50_ values of the four compounds, 2-hydroxy alpinolide has a higher allelopathic potential than the other three compounds. The variations in inhibitory activity of these compounds may result from the variations in structure of phytotoxic substances [50]. In addition, the stronger growth-inhibitory activity of 2-hydroxy alpinolide may be related to a hydroxy group at the C2 position and the furan ring on the structure. Okada et al. (1990) [51] and Wang et al. (2005) [52] noted that natural furans, with the existence of furan and other aromatic rings, have strong biological activity. According to the results of this study, *C. oblongifolius* leaves have their own distinct allelopathic activity, which is affected by the allelochemical compounds (3*R*,6*R*,7*E*)-3-hydroxy-4,7-megastigmadien-9-one, 2-hydroxy alpinolide, alpinolide, and epialpinolide. Additionally, the *C. oblongifolius* tree has abundant leaves, which can be used for living and dead mulch as weed-suppressive or soil-additive resources for natural weed control. Therefore, the allelopathy of *C. oblongifolius* may help to minimize the use of synthetic herbicides to manage weeds as well as ameliorate the adverse effects of the synthetic herbicides on the environment.

## 4. Materials and Methods

### 4.1. Plant Materials

Fresh and mature leaves of *C. oblongifolius* were collected in May 2020 from Khin-U Township, Sagaing Division, Myanmar (22°49′4″ N and 95°48′12″ E) (Figure 1). The leaves were dried in the shade and then ground into a powder using an electric grinder (Cyclotec 1093 sample mill, Tecator AB, Hoedanaes, Höganäs, Sweden). The leaf powder was reserved in plastic bags and maintained at 2 °C for further investigation. Four tested plant species (cress, lettuce, barnyard grass, and timothy) were selected for the bioassays.

### 4.2. Extraction and Bioassay

Leaf powder (300 g DW) was extracted with 2 L of 70% (*v*/*v*) aqueous methanol for 48 h in the dark. The extract was filtered through a sheet of filter paper (No. 2; Toyo Ltd., Tokyo, Japan). After filtration, the remaining residue was re-extracted with methanol (2 L) for 24 h and filtered. The two filtrates were then combined and evaporated at 40 °C under decreased pressure to produce crude extracts. Six different concentrations of 1, 3, 10, 30, 100, and 300 mg DW (milligrams dry weight) equivalent extract/mL were prepared for the bioassay experiment after the crude extracts were dissolved in methanol. The concentrated extract (0.6 mL) was added to sheets of filter paper (No. 2; Toyo Ltd.) in 28 mm Petri dishes. The methanol in each Petri dish was evaporated in a draft chamber. The dried filter paper was then immersed in 0.6 mL of a 0.05% aqueous solution of polyoxyethylene sorbitan monolaurate (Tween 20; Nacalai Tesque, Inc., Kyoto, Japan). Ten sprouted seeds (incubated at 25 °C for 36 to 48 h in the darkness) of barnyard grass and timothy, and ten seeds of cress and lettuce, were placed in the Petri dishes and incubated in the dark at 25 °C for 48 h. Tween 20 solutions without methanol extracts were used as the control treatments. The lengths of the shoots and roots of the examined seedlings were measured and compared with the lengths of the shoots and roots of the control seedlings to determine the percentage of seedling length inhibition. The bioassay experiments were repeated twice using a completely randomized design (CRD) with three replicates and 10 tested plants (per treatment) (*n* = 60).

### 4.3. Isolation and Purification of the Bioactive Substances

**Figure 7:**
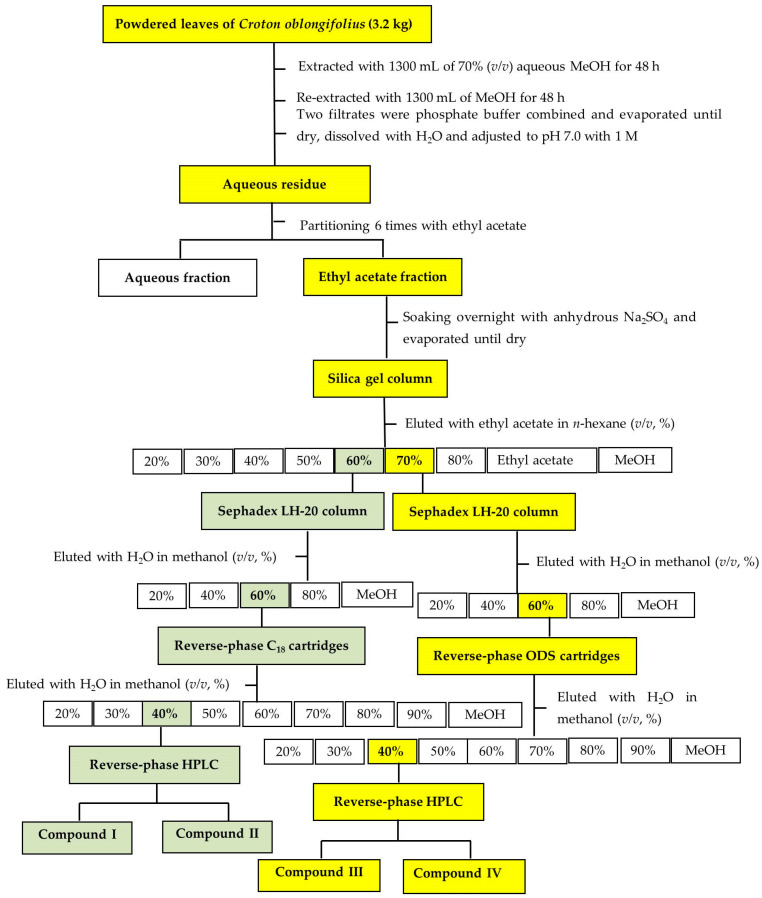
Extraction and purification procedure for four active compounds from *Croton oblongifolius*.

The powdered leaf of *C. oblongifolius* (3200 g DW) was extracted as described in the extraction and bioassay section. The extract was concentrated by using a rotary evaporator to obtain an aqueous residue at 40 °C. The aqueous residue was adjusted to pH 7.0 with 1 M phosphate buffer and divided six times against an equal volume of ethyl acetate (EtOAc). The obtained EtOAc fraction was concentrated to dryness and separated using silica gel chromatography. Of the nine fractions, fractions F5 and F6, eluted with 60 and 70% EtOAc in *n*-hexane, respectively, both showed biological activity (Figure 7). Active fractions F5 and F6 were evaporated, and the residues were separated using a Sephadex LH-20 column. The inhibitory activity of all fractions was determined by a cress bioassay, as mentioned in Section 2.2. The active peak fractions F5 and F6 were eluted with 60% aqueous methanol in Sephadex LH-20.

Active fraction F5 was further separated using an ODS cartridge. The active fraction was eluted with 40% aqueous methanol (Figure 7). After evaporation, F5 was purified using reverse-phase HPLC (3 µm, 4.6 × 250 mm I.D., Inertsil ODS-3; GL Science Inc., Tokyo, Japan; detection at 220 nm wavelength) at a flow rate of 0.5 mL per minute with 45% aqueous MeOH. Inhibitory activity was observed in active peak fractions 1 and 2 at the retention times 54–62 and 64–68 min, respectively, yielding active compounds I and II.

Active fraction F6 was further separated using an ODS cartridge. The active fraction was eluted with 40% aqueous methanol (Figure 7). After evaporation, F6 was purified using reverse-phase HPLC (3 µm, 4.6 × 250 mm I.D., Inertsil ODS-3; GL Science Inc.; detection at 220 nm wavelength) at a flow rate of 0.5 mL per minute with 40% aqueous MeOH. Inhibitory activity was observed in active peak fractions 3 and 4 at the retention times 132–135 and 135–138 min, respectively, resulting in active compounds III and IV. The chemical structures of compounds I, II, III, and IV were identified through analyses of ^1^H and ^13^C NMR spectrums (500 MHz, CDCl_3_ and CD_3_OD) and the specific rotations.

### 4.4. Biological Activity of Compounds I, II, III, and IV

Compounds I, II, III, and IV were diluted by dissolving with 2 mL of MeOH, and six treatment concentrations of 0.01, 0.03, 0.1, 0.3, 1, and 3 mM were prepared. The biological activity of each concentration was examined by a cress bioassay, as described in Section 2.2. The shoot and root length of the cress was measured and the inhibition was determined, as described in the extraction and bioassay section. Each treatment in the bioassay experiments was performed using a CRD design with three replicates and 10 tested plants (per treatment) (*n* = 30).

### 4.5. Statistical Analysis

The bioassay experiments were set using a CRD design with three replications and repeated twice. Data from all ANOVAs were analyzed using SPSS statistical software (version 16.0). Statistically significant variations between the control and treatments were determined using Tukey’s HSD test at the 0.05 probability level. The concentrations necessary for 50% growth reduction (*I*_50_) of each plant species in the bioassays were calculated using Graph Pad Prism Software (Version 6.0).

## 5. Conclusions

The extracts of *C. oblongifolius* exhibited significant phytotoxic potential against the seedling development of both dicot (cress and lettuce) and monocot plants (barnyard grass and timothy). Four compounds (one C_13_ *nor*-isopenoid and three sesquiterpenes) were separated from the *C. oblongifolius* leaves through several purification steps and characterized as (3*R*,6*R*,7*E*)-3-hydroxy-4,7-megastigmadien-9-one, 2-hydroxy alpinolide (novel compound), alpinolide, and epialpinolide through spectral analyses. These identified compounds have a considerable inhibitory effect on cress shoot and root growth. The novel compound 2-hydroxy alpinolide showed the strongest allelopathic effects against cress. The growth-suppressing activities of these compounds may be attributed to the allelopathy of *C. oblongifolius* leaves. However, more field research is required to confirm the allelopathy of *C. oblongifolius* and clarify the mechanism of action of its characterized compounds. Therefore, the leaves (residue) of *C. oblongifolius* might be useful for living and dead mulch as weed-suppressive or soil-additive resources, and its allelopathic compounds could be used to develop an allelochemical-based natural herbicide to decrease the reliance on synthetic herbicides in sustainable agriculture.

## Figures and Tables

**Table 1:** *I*_50_ values for the shoot and root growth of the dicot plants (cress and lettuce) and monocot plants (barnyard grass and timothy) via the aqueous methanol extracts of *Croton oblongifolius*, and their correlation coefficients (r).

Plant Species		*I*_50_ Value (mg DW Equivalent Extract/mL)		Correlation Coefficient (r)	
		Shoot	Root	Shoot	Root
Dicots	Cress	9.4 c	8.3 c	−0.83 ***	−0.81 ***
	Lettuce	5.8 de	8.2 c	−0.89 ***	−0.91 ***
Monocots	Barnyard grass	65.7 a	18.2 b	−0.72 ***	−0.77 ***
	Timothy	7.6 cd	4.8 e	−0.71 ***	−0.74 ***

The letters mean significant difference at less than the 0.05 probability level (Tukey’s test). Asterisks denote statistical significance: *** *p* < 0.001.

**Table 2:** All (^1^H and ^13^C) NMR data for compound II in CD_3_OD.

Position	δ_H_ Mult (*J* in Hz) ^a^	δ_C_^b^
1	2.36, dd (7.5, 3.8)	56.5
2		88.7
3a	2.47, m	36.9
3b	1.66, ddd (13.7, 9.0, 4.9)	
4a	2.03, m	32.6
4b	1.51, m	
5	2.48, m	36.1
6	0.85, d (7.3)	16.0
7		214.1
8	2.35, s	26.9
2’		175.7
3’	5.87, t (1.6)	115.1
4’		182.4
5’	5.41, dd (3.8, 1.8)	83.1
6’	2.71, m	28.6
7’	1.16, d (7.0)	22.2
8’	1.28, d (6.6)	20.3

^a^ Recorded at 500 MHz. ^b^ Quaternary carbon and protonated carbon assignments were established from the HMBC and HSQC spectra, respectively.

**Table 3:** *I*_50_ values (mM) of compounds I, II, III, and IV against the shoot and root growth of cress.

Tested Plant		*I*_50_ Value (mM)			
		Compound I	Compound II	Compound III	Compound IV
**Cress**	Shoot	0.24 a	0.11 c	0.12 c	0.16 b
	Root	0.15 b	0.04 e	0.07 d	0.09 cd

The letters denote a significant difference at less than the 0.05 probability level (Tukey’s test).

## Data Availability

Data will be made available on request.

## Supplementary Materials

The following supporting information can be downloaded at: https://www.mdpi.com/article/10.3390/plants12193384/s1. Figure S1: Full spectrum of ESIMS analysis of compound II; Figure S2: ^1^H NMR spectrum of compound II in CD_3_OD (500 MHz); Figure S3: COSY spectrum of compound II in CD_3_OD (500 MHz); Figure S4: HMBC spectrum of compound II in CD_3_OD (500 MHz); Figure S5: HSQC spectrum of compound II in CD_3_OD (500 MHz).
