# Supplementary material for: Allelopathic Activity of a Novel Compound, Two Known Sesquiterpenes, and a C13 Nor-Isopenoid from the Leave of Croton oblongifolius Roxb. for Weed Control
Source: Plants (Basel). 2023 Sep 25;12(19):3384. doi: 10.3390/plants12193384 (PMC10574435; doi:10.3390/plants12193384)
Supplement: Supplementary file 1 [file plants-12-03384-s001.zip › plants-2589634-supplementary.pdf]

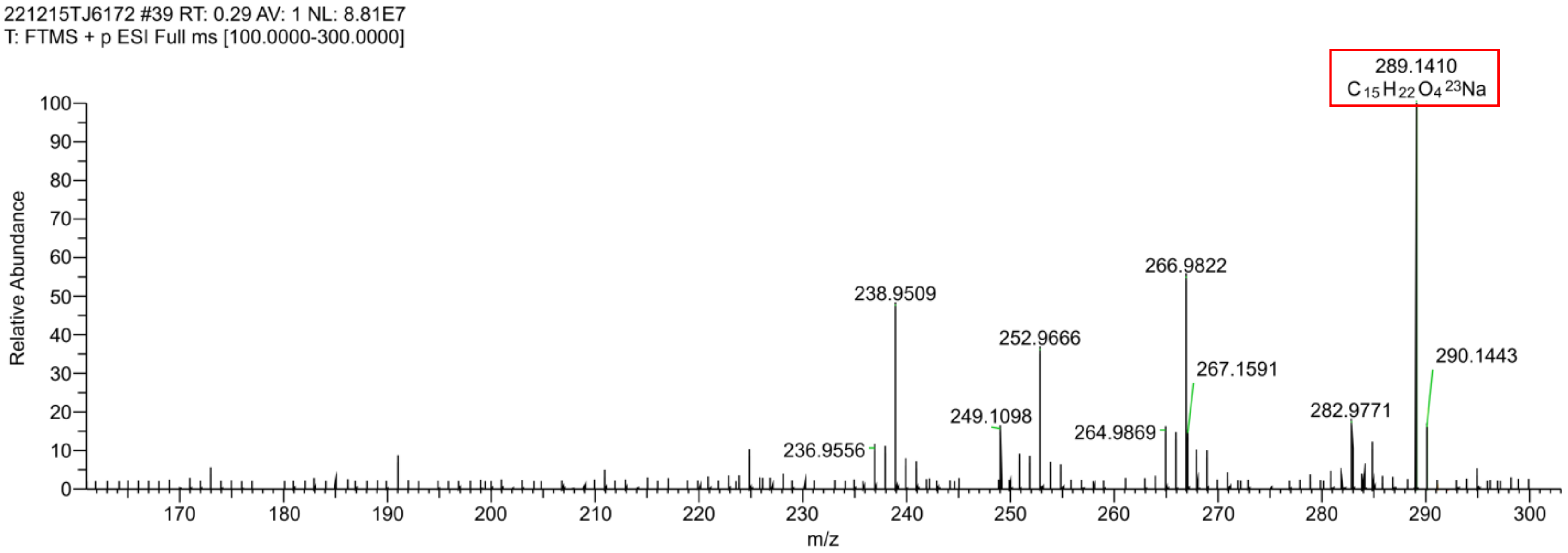

Figure S1. Full spectrum of ESIMS analysis of compound II

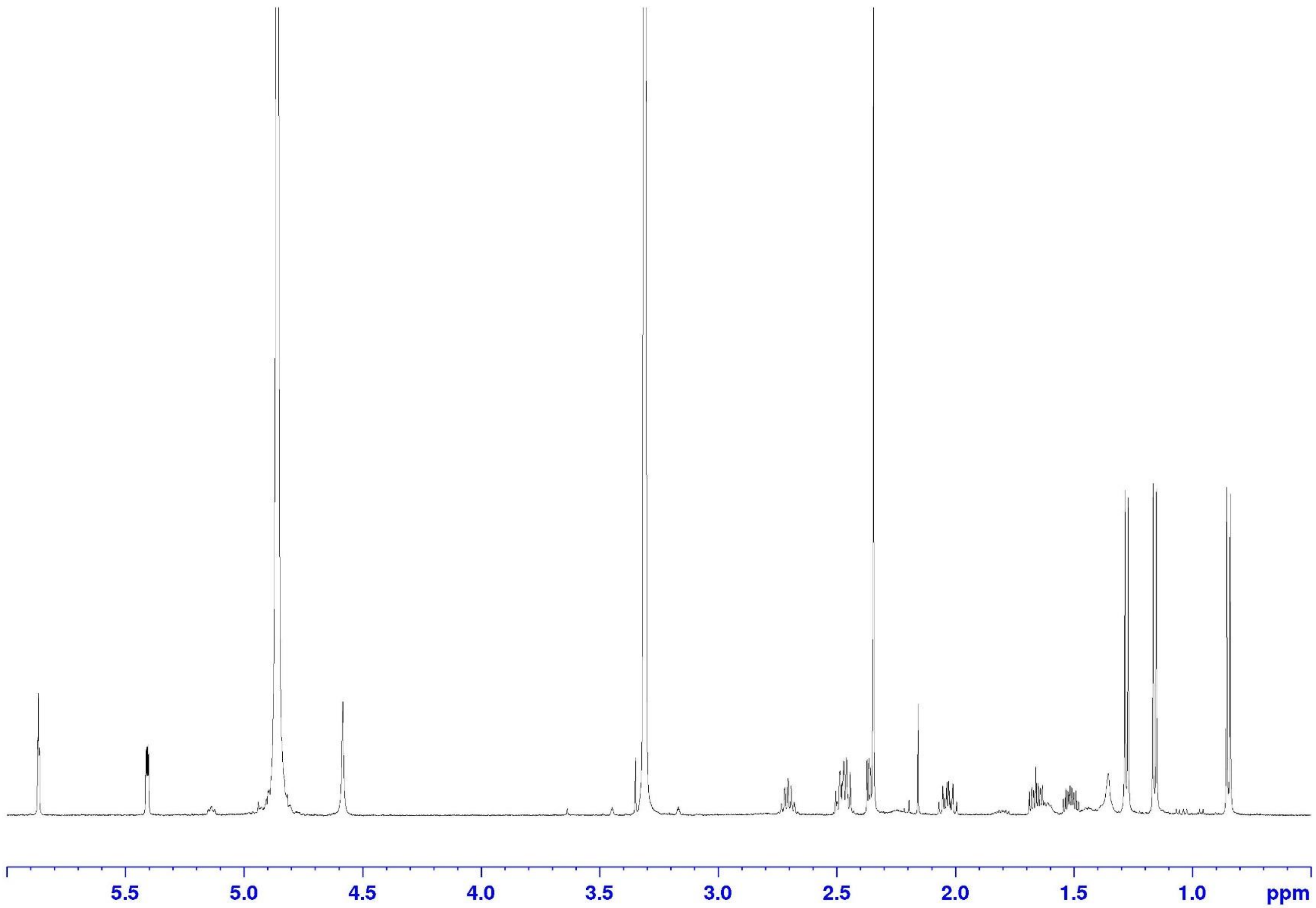

Figure S2. ^{1}H NMR spectrum of compound II in CD_3OD (500 MHz)

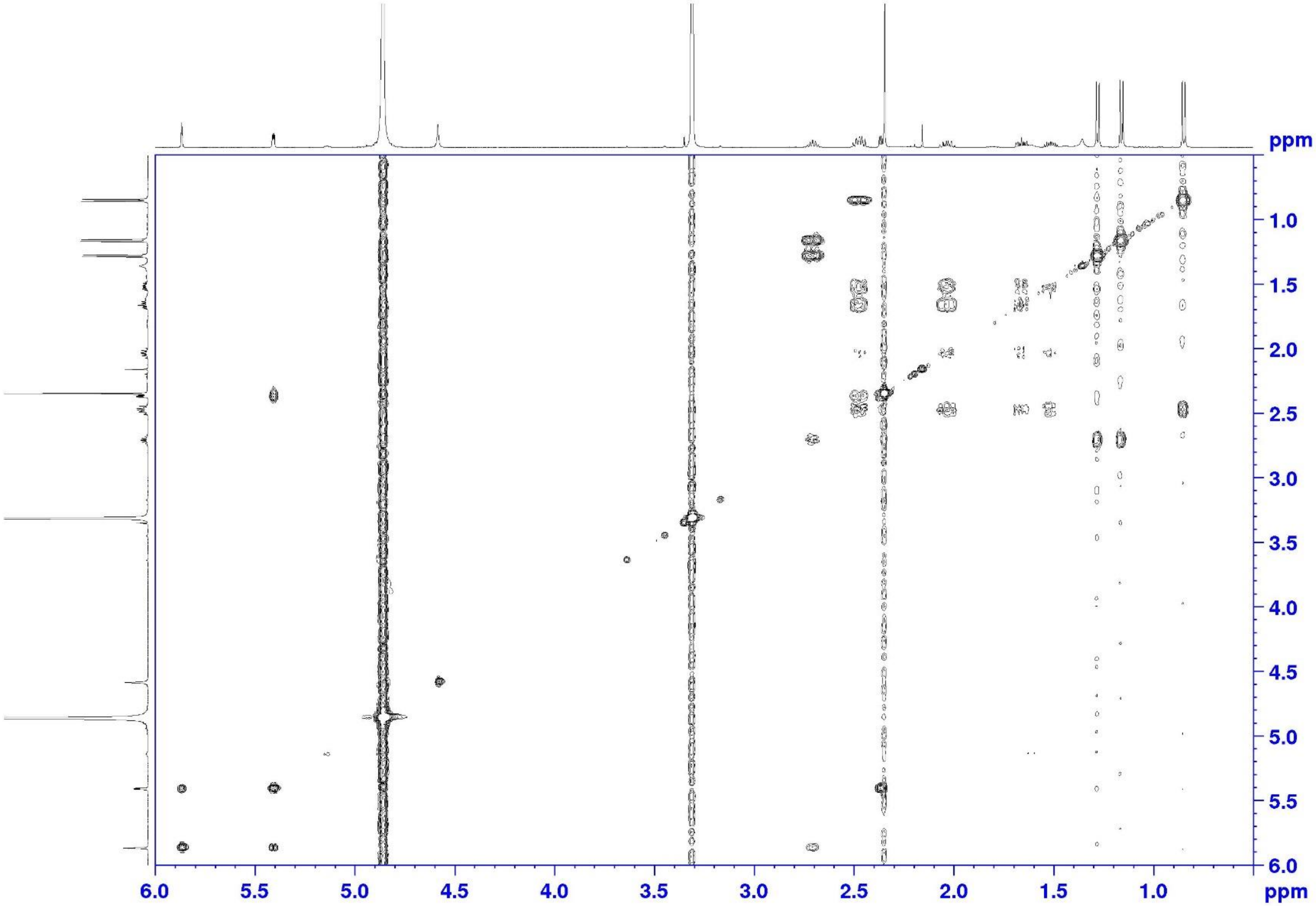

Figure S3. COSY spectrum of compound II in CD_3OD (500 MHz)

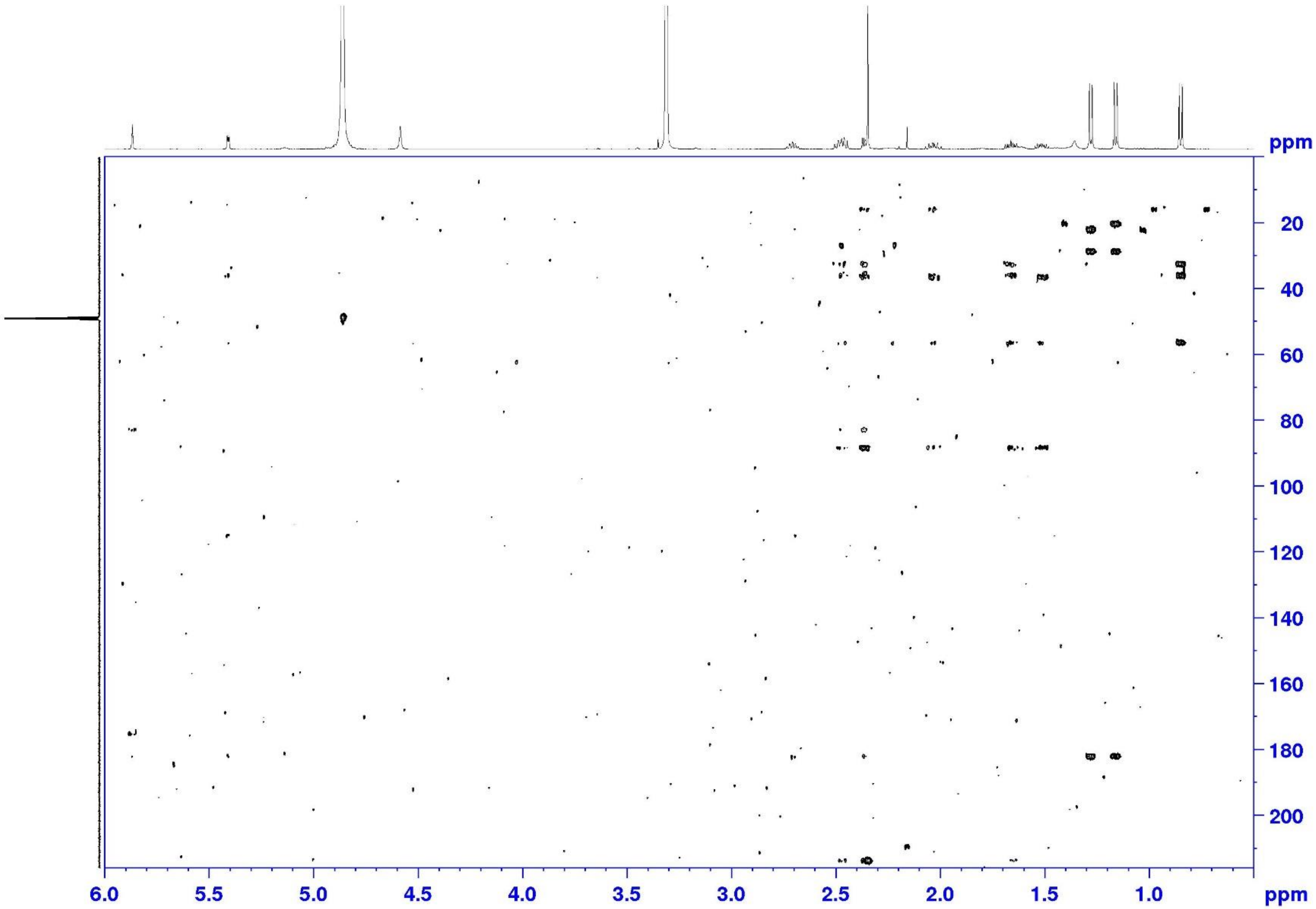

Figure S4. HMBC spectrum of compound II in CD_3OD (500 MHz)

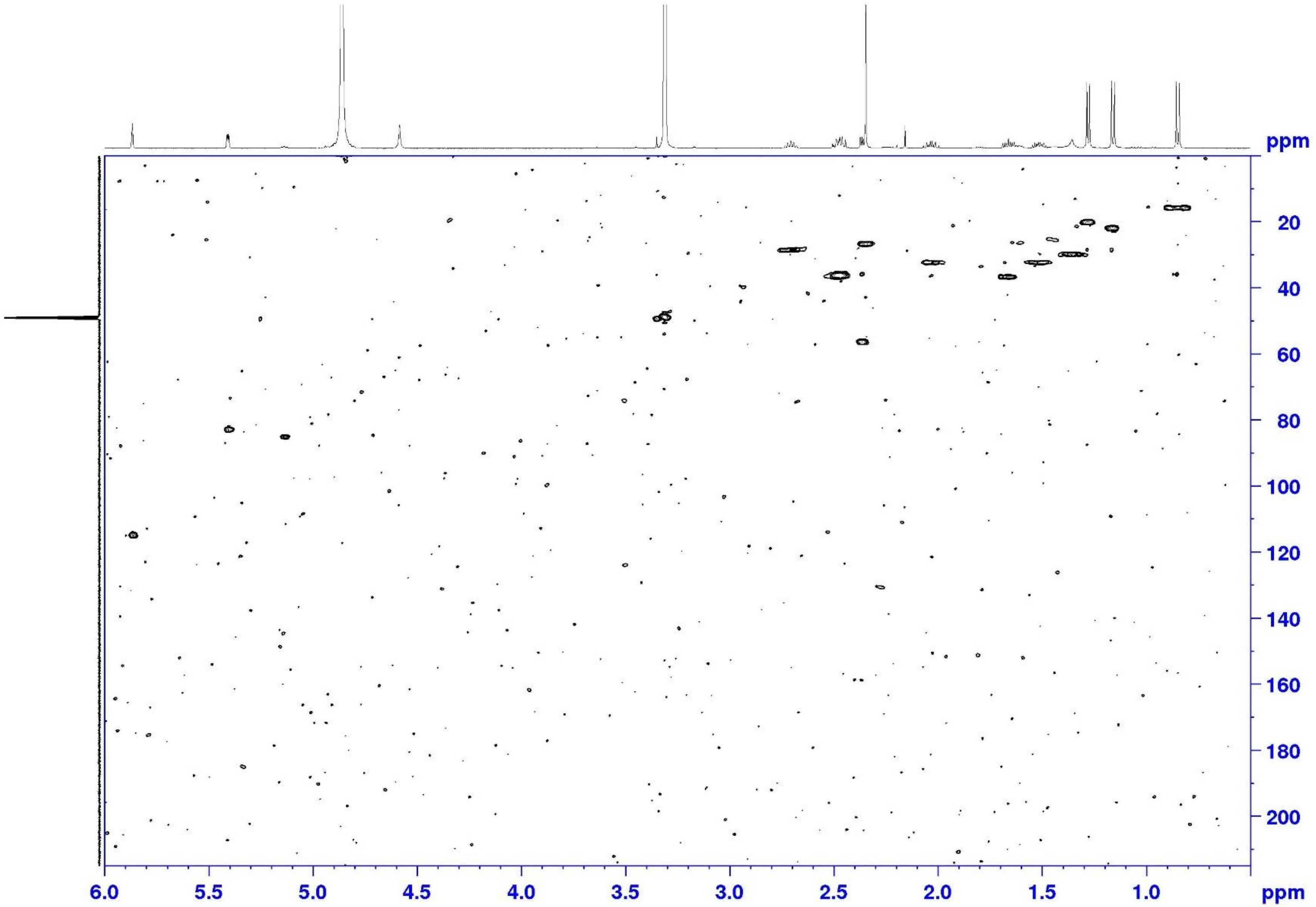

Figure S5. HSQC spectrum of compound II in CD_3OD (500 MHz)
